# Oxidative Balance and Inflammation in Hemodialysis Patients: Biomarkers of Cardiovascular Risk?

**DOI:** 10.1155/2019/8567275

**Published:** 2019-02-11

**Authors:** Daniele La Russa, Daniela Pellegrino, Alberto Montesanto, Paolo Gigliotti, Anna Perri, Antonella La Russa, Renzo Bonofiglio

**Affiliations:** ^1^Department of Biology, Ecology and Earth Sciences, University of Calabria, Italy; ^2^LARSO (Analysis and Research on Oxidative Stress Laboratory), University of Calabria, Italy; ^3^Department of Nephrology, Dialysis and Transplantation, Kidney and Transplantation Research Center, Annunziata Hospital, Cosenza, Italy

## Abstract

During chronic kidney disease, the progressive deterioration of renal function induces several biological/clinical dysfunctions, including enhancement of synthesis of inflammation/oxidative stress mediators. Impaired renal function is an independent cardiovascular risk factor; indeed, cardiovascular complications dominate the landscape of both chronic kidney disease and end-stage renal disease. The aim of this study is to explore the correlation between the global oxidative balance in hemodialysis patients and both inflammatory markers and cardiovascular events. Using photometric tests, this study explored plasmatic oxidative balance in 97 hemodialysis patients compared to a healthy population. In the hemodialysis patients, we showed that oxidative stress values were significantly lower than in controls while effectiveness in the antioxidant barrier was significantly increased in the hemodialysis group. Furthermore, we highlighted a strong correlation between oxidative index and blood levels of C-reactive protein. When patients were divided into two groups based on previous cardiovascular events, we found that subjects with previous cardiovascular events had higher values of both oxidative stress and antioxidant barrier than patients without cardiovascular events. Our results indicated that in hemodialysis patients, the clinical and prognostic significance of oxidative status is very different from general population. As cardiovascular complications represent a strong negative factor for survival of hemodialysis patients, the research of new cardiovascular risk biomarkers in these patients takes on particular importance in order to translate them into clinical practice/primary care.

## 1. Introduction

Chronic kidney disease (CKD) is a major public health problem worldwide, and its main consequences include loss of renal function leading to end-stage renal disease (ESRD), increased risk of cardiovascular disease (CVD), significant increase in morbidity and mortality, and a decrease in health-related quality of life [1–3]. A recent meta-analysis of observational studies reported that CKD has a high global prevalence (between 11 and 13%) with percentage prevalence higher in developed areas such as Europe, USA, Canada, and Australia, where the populations of elderly is greater than in developing areas [4]. Indeed, the risk of CKD increases with age and elderly patients are overrepresented in the dialysis population [5]. However, there seems to be a complex relationship between aging and CKD, as geriatric complications are highly detectable in younger patients with ESRD [6]. This has led to the hypothesis of a premature biological aging process of different organ systems associated with CKD [7].

During CKD, the progressive deterioration of kidney function has important systemic effects due to its central role in body homeostasis and induces several biological and clinical dysfunctions including alteration in cellular energetic metabolism, change in nitrogen input/output, protein malnutrition, resistance to insulin, and considerable enhancement of synthesis of inflammation/oxidative stress mediators. Inflammatory markers such as C-reactive protein and cytokines increase with renal function deterioration suggesting that CKD is a low-grade inflammatory process [8] comparable to the “inflammaging” phenomenon, in which aging tissues exhibit low-grade, chronic, systemic inflammation, in the absence of overt infection [9]. A number of factors can be involved in triggering the inflammatory process including oxidative stress. Several authors have reported that in CKD, even in the early stage, there is an abundant production of reactive oxygen species and hemodialysis exacerbates oxidative stress [10]. However, it remains unclear at which stage of renal insufficiency the redox imbalance becomes more profound [11]. Knowledge on causes and consequences of oxidative stress in CKD is rapidly expanding [12], but the factors influencing the oxidative status have not been characterized in these patients as well as the prognostic importance of circulating oxidative stress biomarkers remains poorly understood.

Impaired renal function is an independent cardiovascular risk factor; indeed, cardiovascular complications dominate the landscape of both CKD and ESRD [13]. The complex relationship of CKD with CVD is likely due to both traditional (age, hypertension, diabetes, and dyslipidemia) and CKD peculiar risk factors (volume overload, mineral metabolism abnormalities, proteinuria, malnutrition, oxidative stress, and inflammation). CKD patients can display opposite associations with traditional CVD risk factors as obesity, hypercholesterolemia, and hypertension that paradoxically appear to be protective features, in contrast to the general population [14]. Indeed, death associated with these pathologies cannot be attributed only to complications of atherosclerotic disease (myocardial infarction, stroke, and heart failure), but other specific processes contribute to cardiovascular morbidity and mortality in these patients [15]. As traditional risk factors are poor predictors in patients with late-stage CKD, identifying and intervening against new risk factors are priorities.

The aim of this study is to explore the global oxidative balance in ESRD hemodialysis (HE) patients compared to a healthy population. Since inflammatory stress and cardiovascular complications represent two very frequent conditions in hemodialysis subjects, the correlation between the oxidative parameters and both inflammatory markers and cardiovascular events was also investigated.

## 2. Methods

### 2.1. Subjects

The study involved 97 patients older than 18 years of both sexes with ESRD on regular hemodialysis therapy for at least 6 months (HD, mean age 67.25 ± 1.66) recruited from the Department of Nephrology, Dialysis and Transplantation, Kidney and Transplantation Research Center, Annunziata Hospital of Cosenza (Italy), between August 2017 and December 2017. The hemodialysis prescription consisted of 4 hourly, thrice weekly sessions, using polysulfone membrane and bicarbonate-buffered dialysate. The blood flow rate was 300 mL/min and low molecular weight heparin was used as anticoagulant. Hemodialysis adequacy was determined by using Kt/V formula.  Table 1 reports the biochemical profiles of hemodialysis patients.

As controls for patient group, a recruitment campaign focused on the staff of the University of Calabria within the frame of the prevention and protection program adopted by the same university. In particular, 95 healthy volunteers age- and sex- matched with the ESRD group (CTR, mean age 63.7 ± 0.33) were recruited between August 2017 and December 2017. They underwent through a structured interview during which sociodemographic, anthropometric, and clinic information was collected. The lifestyle was evaluated using self-administered questionnaires, which included items on smoking, exercise habits, and drinking habits. Anthropometric measurements included measurements of weight and height. Body mass index (BMI) was calculated as weight (kg)/height (m^2^), while blood pressure was measured in the sitting position using an automatic sphygmomanometer (Microlife BP A2). The biochemical measurements included glucose, total cholesterol, triglyceride (TG), high-density lipoprotein (HDL), low-density lipoprotein (LDL), HbA1c, uric acid (UA), and creatinine levels. Blood tests also included the evaluation of prooxidant, and antioxidant status. Exclusion criteria included a family history of ESRD or an estimated glomerular filtration rate (eGFR) lower than 60 mL/min/1.73 m^2^, calculated by CKD-EPI equation [16].

All subjects (patients and controls) were studied in the morning and in a fasting state. Blood samples were taken from the antecubital vein and immediately centrifuged (2500 g for 15 min at 4°C), and the plasma obtained was stored at 4°C until measurements (maximum 6 hours of venous blood collection). Standard blood tests were performed at the clinical laboratory of Annunziata Hospital, Cosenza; prooxidant and antioxidant status were determined at the Analysis and Research on Oxidative Stress Laboratory (LARSO) of the University of Calabria.

### 2.2. Oxidative Status and Biological Antioxidant Potential Measurements

Oxidative stress and biological antioxidant potential determination were performed by using photometric measurement kits and a free radical analyzer system provided with spectrophotometric device reader (FREE Carpe Diem, Diacron International, Grosseto, Italy). The d-ROM test helps to determine the oxidant ability of a plasma sample measuring the presence of reactive oxygen metabolite derivatives, in particular, hydroperoxides (oxidative index). Results are expressed in Carratelli units (UC; 1 UC = 0.8 mg/L of hydrogen peroxide). The BAP test provides an overall measure of the biological antioxidant potential measuring the blood concentration of antioxidants (such as bilirubin, uric acid, vitamins C and E, and proteins) capable of reducing the iron from ferric to the ferrous form (antioxidant barrier). Results are expressed in *μ*mol/L of the reduced ferric ions.

### 2.3. Statistical Analysis

Differences between groups were examined using the independent sample *t*-test. The correlation between continuous variables was assessed using Pearson's coefficient. Data have been analyzed using R statistical language (http://www.r-project.org/).

## 3. Results

Our study population consists of 97 hemodialysis patients and 95 healthy volunteers as control group.  Table 2 reports the demographic characteristics of the analyzed samples together with the oxidative and inflammatory parameters and CVD prevalence.

**Figure 1:**
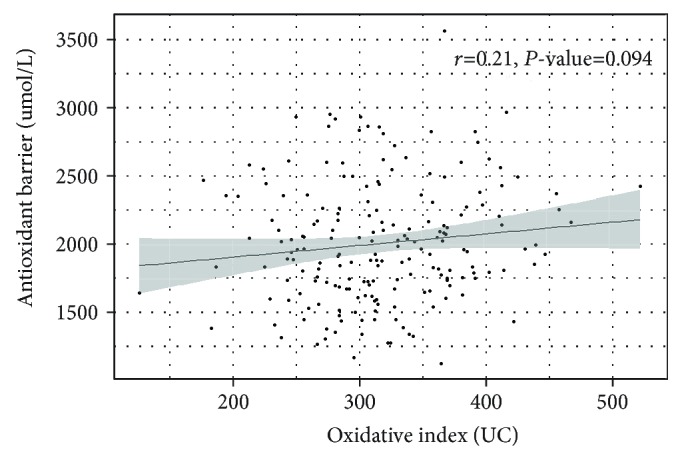
Correlation between oxidative index and antioxidant barrier values in HD patients (*r* = Pearson correlation coefficient).

We found that in the HD sample, the oxidative index was significantly lower than in CTR (304.25 vs 332.4, *P* value = 0.002). Moreover, ESRD patients also showed a greater effectiveness in the antioxidant barrier than CTR subjects (2127.4 vs 1888.39, *P* value = 1.24^∗^10^−5^). The relationship between oxidative index and antioxidant barrier values showed a borderline correlation (*r* = 0.121; *P* value = 0.094; Figure 1). In healthy subjects, literature data showed significant gender and age differences in oxidative stress biomarkers [17]. In our hemodialysis patients, by linear regression analysis, we verified that these results are not related to both age and sex.

**Figure 2:**
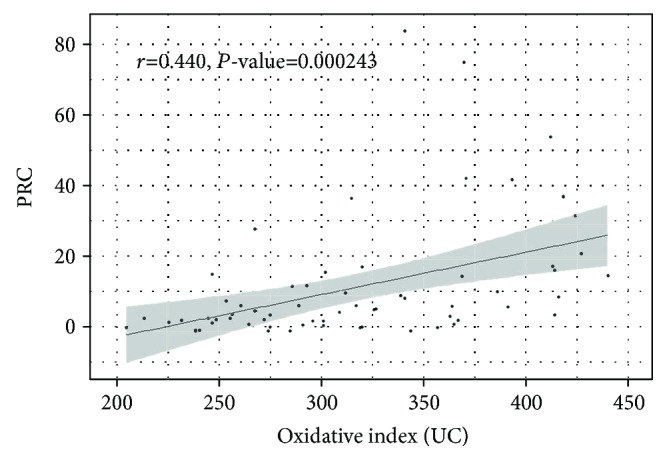
Correlation between oxidative index and PCR (C-reactive protein) values in HD patients (*r* = Pearson correlation coefficient).

It is well known that CKD subjects present a significant increase of both inflammation and oxidative stress mediators. In our HD group, we found a strong correlation between oxidative index and blood levels of C-reactive protein (*r* = 0.440; *P* value = 2.43^∗^10-5; Figure 2), while antioxidant barrier efficacy was not correlated with this inflammatory marker.

**Figure 3:**
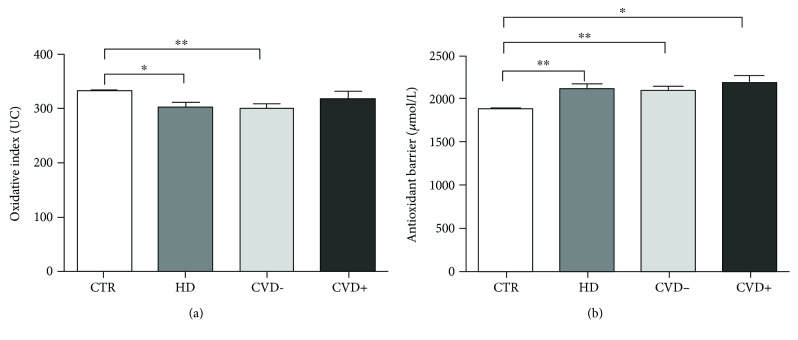
Values of d-ROM (a) and BAP (b) tests in a healthy population (CTR), all hemodialysis patients (HD), hemodialysis patients without cardiovascular events (CVD-), and hemodialysis patients with previous CVD events (CVD+). Data are expressed as mean±SE (Tukey's multiple comparison test, ^∗^*p* < 0,005; ^∗∗^*p* < 0, 0005).

To explore the putative relationship between oxidative status and classic cardiovascular risk factors in ESRD, linear regression analysis was performed. In particular, we observed that gender, diabetes, age, and lipid profile (total cholesterol and LDL and HDL cholesterol) were not related to both oxidative index and antioxidant barrier efficacy. Concerning smoking habits, it has not been possible to analyze any correlations given the small number of smoking subjects. Interestingly, when HD patients were stratified according to previous CVD events, we detected that subjects with previous acute myocardial infarction (CVD+, *n* = 18) had higher values of both oxidative stress and antioxidant barrier than patients without cardiovascular events (CVD-; *n* = 79; Figures 3(a) and 3(b)). It is remarkable to note that HD subjects with previous CVD events had oxidative stress values comparable to healthy controls while the efficacy of the antioxidant barrier was considerably and significantly enhanced compared to all the groups.

## 4. Discussion

Our research focused on the evaluation of the overall redox state on a significant hemodialysis population. Our results clearly showed that the hemodialysis patients presented oxidative stress values significantly lower and antioxidant barrier effectiveness significantly higher compared to healthy controls. Despite the good plasmatic redox status found in all hemodialysis patients, we highlight a strong correlation between oxidative index and C-reactive protein blood levels. In addition, we reported that, in our cohort of hemodialysis patients, subjects with previous acute myocardial infarction had higher values of both oxidative stress and antioxidant barrier respect to patients without cardiovascular events. It is noteworthy that these results have been realized using noninvasive method to detect total plasma redox balance, which is of particular importance when analyzing the frail hemodialysis patients. Indeed, to detect plasmatic redox status, we utilized two simple methods widely used in recent years, the d-ROM and the BAP tests [17–20]. The predictive ability of these biomarkers and their usefulness in cardiovascular and renal disease has been tested by our research group on both human and mammalian models [21–23].

Several authors reported a profound imbalance between oxidants and antioxidants in CKD [24]. The balance between pro- and antioxidant systems is essential for the regular function of organism's cells and molecules. Transiently increased concentrations of reactive oxygen species (ROS) perform specific physiological roles in keeping the organism's homeostasis in the redox-related signaling and also in the immune defense system, as they are produced in high amounts in inflammation [12, 25, 26]. Since CKD is characterized by a steady, low-grade inflammation, the long-lasting high ROS levels can lead to oxidation of DNA, lipids, and proteins with consequent cellular damage in CKD patients [12]. In addition, an impaired renal function leads to the accumulation of toxins and waste metabolites inducing an imbalance in redox homeostasis [11]. However, it remains unclear at which stage of renal insufficiency the redox imbalance becomes more profound and if dialytic treatment increases redox imbalance [11, 27]. Studies on various plasma and erythrocyte parameters of oxidative stress (free radicals, ROS, and lipid peroxidation products) and antioxidant defense have shown conflicting results [24]. Dursun and coworkers [28] described a high oxidative stress and an impaired antioxidant response in both hemodialysis and predialysis uremic patients. These authors ascribed in part this redox imbalance to antioxidant enzyme deficiency, but in parallel, they found an increased protein carbonyl content, an indicator of oxidative protein damage, in predialysis patients but not in hemodialysis subjects thus supporting the hypothesis of high oxidative stress due to uremic state [28].

In our cohort of hemodialysis patients, we detected oxidative stress values significantly lower compared to healthy controls. As index of oxidative stress, we did not analyze the oxidative damage of single macromolecules but the plasmatic oxidant ability by measuring the presence of reactive oxygen metabolite derivatives, in particular, hydroperoxides. Actually, the most direct approach for lipid peroxidation evaluation is the quantification of the primary products, hydroperoxides, rather than the secondary or end products derived from hydroperoxides such as malondialdehyde. Plasmatic hydroperoxides are a sensitive and specific index that the presence of oxidative stress in vivo providing a global assessment of the imbalance induced by the alteration of the individual parameters [21]. Indeed, various serum markers of lipid and protein oxidation have been tested for the evaluation of oxidative stress in vivo, but the contrasting results obtained clearly indicate that a single marker does not have the ability to indicate the real redox state of the subjects [28–31].

Concerning the antioxidant systems, literature data are really complex to discern. Most of the studies in CKD patients have analyzed expression/activity of antioxidant enzymes with conflicting results [11, 27]. In addition, most of the reports concentrate on a single or a few antioxidants. The stability/activity of enzymatic antioxidant systems is multifaceted: in the case of low/medium oxidative stimulation, enzymatic antioxidant activity can increase, but if oxidative stress is persisting, or its level is very high, the damage caused to proteins becomes profound and a decreased expression/activity may occur via direct oxidative damage of the molecules and/or oxidative-altered gene expression. An alternative approach to investigate the antioxidative defense is the assessment of nonenzymatic ascorbate, glutathione, flavonoids, tocopherols, and carotenoids [32]. Unlike antioxidant enzymes, the ROS stimulation can determine a depletion of nonenzymatic antioxidants since the ROS species neutralization implies their consumption [33]. Interestingly, our results clearly showed that the hemodialysis patients presented a greater effectiveness in the nonenzymatic antioxidant barrier respect to healthy controls. This could be explained by the fact that we have measured the plasma total antioxidant capacity and not a single antioxidant specie. Since the antioxidant species are numerous and they operate synergistically, evaluating the activity of each antioxidant species may underestimate the association among different effects and probably do not reflect the physiological conditions [34]. Furthermore, cellular antioxidants are under homeostatic control and a decrease in a particular antioxidant can be compensated by an increase in a different one [35, 36]. Our results contribute to emphasize the importance of total antioxidant barrier effectiveness in countering the deleterious effects of ROS overproduction in hemodialysis patients, as suggested by several authors who reported antioxidant therapy as an early intervention to stop premature cardiovascular disease in CKD [12].

CKD is a low-grade, chronic, systemic inflammation with considerable enhancement of synthesis of inflammation mediators such as C-reactive protein and cytokines [8]. Literature data showed a significant increase of plasmatic C-reactive protein also in pediatric patients on hemodialysis [37], and this continuous inflammatory status is related to adverse outcomes mainly for cardiovascular events [7]. A number of factors can be involved in triggering the inflammatory process including oxidative stress [7, 29]. Despite the good plasmatic redox status found in our hemodialysis patients, we highlight a strong correlation between oxidative index and C-reactive protein blood levels, confirming that the inflammatory status is an important factor relating to oxidative stress in hemodialysis patients.

In recent decades, numerous studies and meta-analyses have highlighted that impaired renal function is an independent cardiovascular risk factor; indeed, cardiovascular complications dominate the landscape of both CKD and ESRD [13]. Traditional cardiovascular risk factors are poor predictors in patients with late-stage CKD; indeed, death associated with these pathologies cannot be attributed only to complications of atherosclerotic disease (myocardial infarction, stroke, and heart failure), but other processes peculiar to CKD can contribute to cardiovascular morbidity and mortality in these patients [15]. In our study, we detected that patients with previous acute myocardial infarction had higher values of both oxidative stress and antioxidant barrier respect to HD patients without cardiovascular events. This result is of a particular significance as it shows that in HD patients, the “normal range” of oxidative stress levels (calculated in healthy subjects) is a signal of cardiovascular risk. Interestingly, the HD patients with previous cardiovascular event also have an enhanced antioxidant barrier, considerably and significantly higher than all other groups. The underlying mechanism for this plasmatic antioxidant capacity increases was not investigated in the present study; we speculate that there may be a protective compensatory response as already reported in the CKD for the enzymatic antioxidant component [38, 39].

## 5. Conclusions

Our results suggest that in HD patients, the clinical and prognostic significance of oxidative status associated with cardiovascular risk factors is very different from the general population. Although a direct causality cannot be inferred from such kind of correlative investigations, our data provide an important contribution to understanding the cross-talk between oxidative imbalance and cardiovascular risk in CKD also representing a point of departure to address further investigation. As cardiovascular complications represent a strong negative factor for hemodialysis subject survival, the preservation of an optimal global redox balance through effective methods may be considered as a potential target for therapies aimed at preventing cardiovascular complications during CKD progression.

## Figures and Tables

**Table 1:** Biochemical profiles of the hemodialysis patient cohort.

	Mean±SE
Albumin (g/dl)	6.567 ± 3.27
Cholesterol, total (mg/dl)	154.9 ± 4.14
HDL cholesterol (mg/dl)	43.65 ± 1.42
LDL cholesterol (mg/dl)	79.38 ± 3.88
C reactive protein (mg/dl)	12.06 ± 2.10
Uric acid (mg/dl)	5.73 ± 0.16
Urea (mg/dl)	64.73 ± 2.55
Iron, total (*μ*g/dl)	68.53 ± 4.83
Transferrin (mg/dl)	150 ± 5.17
Ferritin (ng/mL)	509.1 ± 41.46
Fibrinogen (mg/dL)	397.6 ± 22.39
Insulin (*μ*IU/mL)	26.65 ± 4.19
White blood cell (106/mL)	6.595 ± 0.25
Platelets (106/mL)	192.1 ± 9.4
Protein, total (g/dL)	6.01 ± 0.12

**Table 2:** Demographic characteristics and redox profile of the cohort.

	Control group	Hemodialysis patients	*P* value
Age (mean±SE)	63.737 ± 0.33	67.25 ± 1.66	0.0355
Oxidative index (UC)	332.4 ± 5.74	304.25 ± 6.66	0.002
Antioxidant barrier (*μ*mol/L)	1888 ± 39.14	2127.4 ± 46.77	1.24^∗^10^−5^
Cardiovascular events (*n*, %)	—	18 (18.5%)	*—*

## Data Availability

The hematochemical data used to support the findings of this study are included within the article.
